# Mechanisms of peripheral phylogeographic divergence in the indo-Pacific: lessons from the spiny lobster *Panulirus homarus*

**DOI:** 10.1186/s12862-017-1050-8

**Published:** 2017-08-18

**Authors:** Ahmad Farhadi, Andrew G. Jeffs, Hamid Farahmand, Thankappan Sarasam Rejiniemon, Greg Smith, Shane D. Lavery

**Affiliations:** 1 School of Biological Sciences, University of Auckland, Auckland, New Zealand; 2 Department of Natural Resources and Environment, School of Agriculture, Shiraz University, Shiraz, Iran; 3 Institute of Marine Science and School of Biological Sciences, University of Auckland, Auckland, New Zealand; 4 Department of Fisheries and Environment, Faculty of Natural Resources, University of Tehran, Tehran, Iran; 5 Department of Botany and Biotechnology, AJ college of Science and Technology, Kerala, India; 6 Institute for Marine and Antarctic Studies, University of Tasmania, Hobart, TAS Australia; 7 School of Biological Sciences and Institute of Marine Science, University of Auckland, Auckland, New Zealand

**Keywords:** Indo-West Pacific, Larval dispersal, Marine biogeography, mtDNA, Microsatellites, *Panulirus*, Peripheral speciation, Phylogeography, West Indian Ocean

## Abstract

**Background:**

There is increasing recognition of the concordance between marine biogeographic and phylogeographic boundaries. However, it is still unclear how population-level divergence translates into species-level divergence, and what are the principal factors that first initiate that divergence, and then maintain reproductive isolation. This study examines the likely forces driving population and lineage divergences in the broadly-distributed Indo-Pacific spiny lobster *Panulirus homarus*, which has peripheral divergent lineages in the west and east. The study focuses particularly on the West Indian Ocean, which is emerging as a region of unexpected diversity. Mitochondrial control region (mtCR) and COI sequences as well as genotypes of 9 microsatellite loci were examined in 410 individuals from 17 locations grouped into 7 regions from South Africa in the west, and eastward across to Taiwan and the Marquesas Islands. Phylogenetic and population-level analyses were used to test the significance and timing of divergences and describe the genetic relationships among populations.

**Results:**

Analyses of the mtCR revealed high levels of divergence among the seven regions (Ф_ST_ = 0.594, *P <* 0.001). Microsatellite analyses also revealed significant divergence among regions, but at a much lower level (F_ST_ = 0.066, *P <* 0.001). The results reveal different patterns of mtCR v. nDNA divergence between the two distinct peripheral lineages: a subspecies in South Africa and Madagascar, and a phylogeographically diverged population in the Marquesas. The results also expose a number of other more fine-scale population divergences, particularly in the Indian Ocean.

**Conclusions:**

The divergence of peripheral lineages in the west and east of the species’ range appear to have been initiated and maintained by very different processes. The pattern of mitochondrial and nuclear divergence of the western lineage, implicates processes of parapatric isolation, secondary contact and introgression, and suggests possible maintenance through adaptation and behavioural reproductive isolation. In contrast, the eastern lineage appears to have diverged through a rare colonisation event, maintained through long-term isolation, and matches expectations of the core-periphery hypothesis. The process of active peripheral speciation may be a common force in the Indo-Pacific that helps drive some of the regions’ recognized biogeographic boundaries.

**Electronic supplementary material:**

The online version of this article (doi:10.1186/s12862-017-1050-8) contains supplementary material, which is available to authorized users.

## Background

Our understanding of the origins of marine biodiversity has been greatly enhanced through investigation of the evolutionary history of biogeographic provinces. This is particularly so in the vast Indo-Pacific region, which harbours the bulk of marine diversity, and has long been of great biogeographic interest across a wide taxonomic range of species [1–5]. Marine biogeographic provinces are largely defined by distributions of species and/or endemism [6, 7]. However, there is increasing recognition of the concordance between marine biogeographic and phylogeographic boundaries [8, 9], and there have been a number of within-species genetic studies that have greatly expanded our understanding of some of the common evolutionary patterns in the Indo-Pacific [10, 11]. However, it is still unclear how population-level divergence translates into species-level divergence [8], and what the differing factors are that first initiate that divergence, and then maintain reproductive isolation [12, 13]. This is particularly puzzling in the marine environment, where many species have the ability to disperse widely through planktonic larvae [14].

Although there are common biogeographic and phylogeographic boundaries among Indo-Pacific species that hint at common causes of divergence, there are also many discrepancies in the patterns observed among species [15, 16]. These discrepancies suggest that a diversity of forces act on marine species in this region to initially create genetic and biogeographic boundaries. These may include not only the obvious restrictions to dispersal of drifting larvae by the prevailing ocean currents [14, 17], but also the impact on both currents and habitat availability by recent sea-level changes, or more distant geological events [18].

It is also unclear what the forces are that maintain reproductive isolation as diverged populations become species, and what is the relative importance of physical versus ecological boundaries in maintaining that divergence [19]. That is, how is it that the dynamic processes of gene flow reduction and population subdivision within species may ultimately lead to biogeographic divergence among species. Although it may at first appear to be mediated in a simple way through allopatric speciation, the complex overlapping distributions of most marine taxa through the region show that the process is far from simple, and have led some to invoke the potential impact of adaptation to differing environments [20].

Another major gap in our understanding of Indo-Pacific biodiversity is the fine-scale pattern of population substructure across the region. The broad biogeographic patterns and provinces are now somewhat clear [8], but there is still much doubt about the locations of less obvious barriers to gene flow within each province, and if these too may be common among species [21]. Such fine-scale questions are of particular importance for management of commercial or endangered species, and require much more intensive sampling than common in past studies across this vast region, especially within the less-studied Indian Ocean, which appears to harbour unrecognised levels of biodiversity [15, 22].

**Fig. 1:**
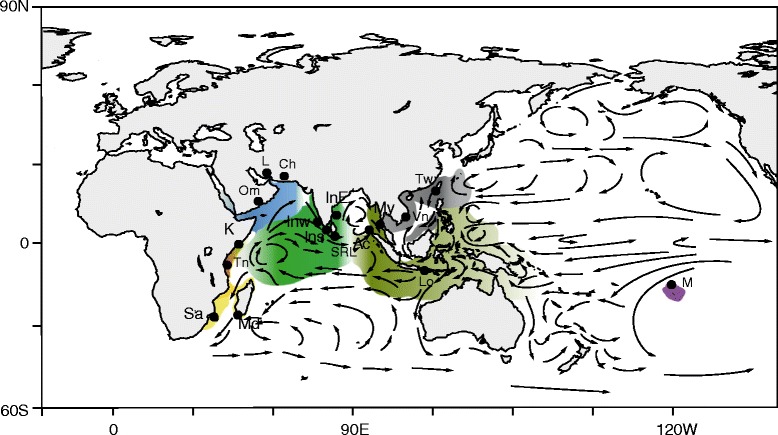
Sampling locations and species distribution of *Panulirus homarus*, with major ocean currents. Regional groupings are shown in colours that are used in subsequent figures. Location abbreviations: *P. h. rubellus* Region: Sa - South Africa, Md - Madagascar; East Africa Region: Tn - Tanzania, K - Kenya; North-West Indian Ocean (NWIO) Region: Om - Oman, Mirbat, L - Iran-Larak Island, C - Iran-Chabahar; India Region: Inw -India west, Ins – India south, InE - India east; Indonesia Region: Ac - Aceh, My - Langkawi, Lo - Lombok; East Asia Region: Vn - Vietnam, Tw - Taiwan; Marquesas Region: M - Marquesas Islands

In this study, we begin to address these outstanding questions using the scalloped spiny lobster, *Panulirus homarus* (Linnaeus, 1758). *P. homarus* is a valuable fisheries species distributed widely across the Indo-Pacific region (Fig. 1) [23]. This species has ocean-going larvae with a larval period (PLD) of at least 6 months [24], which could be expected to provide high levels of genetic connectivity over large spatial scales [14]. Recent mitochondrial and nuclear DNA sequence analyses of small numbers of *P. homarus* across the range of this species [25] revealed three evolutionary lineages that matched three of the four previously described morphological subspecies: *P. homarus rubellus* (in South Africa and Madagascar) [26, 27], *P. homarus “brown”* (in the Marquesas Islands, Central Pacific) [28], and *P. homarus homarus*, which appears to inhabit all the intervening part of its distribution (see Fig. 1). There was found to be no genetic basis for the previously described *P. homarus megasculpta* subspecies [25]. *P. homarus* thus appears to show peripheral endemism, which is now becoming recognised as a common process driving the generation of marine biodiversity [15, 19, 29, 30]. One corollary of peripheral endemism is articulated in the “core-periphery” (or “central-marginal”, or “centre of origin”) hypothesis [31], which predicts that peripheral populations will exhibit lower diversity and greater divergence, a pattern seen in several marine species [32–34].

We investigate here the process of phylogeographic divergence in this species across biogeographic boundaries, to shed light on the evolutionary forces driving this. Using a greatly expanded data set that includes substantially increased sample sizes from a much wider diversity of locations, examined using two mitochondrial DNA genes and nine nuclear microsatellite loci, we address two groups of related questions: (1) What can multiple loci tell us about the processes driving divergence at the western and eastern peripheries? When did the divergences occur, have they both been driven and maintained by similar allopatric processes, do they match the predictions of the core-periphery hypothesis, and are they concordant with those of other species? (2) Do more fine-scale divergences exist between populations separated by other recognised biogeographic boundaries, the Indo-Pacific barrier (IPB), and the proposed mid-Indian Ocean barrier (MIOB [22]). Can we help clarify the nature and location of the MIOB [35].

## Methods

### Sampling, PCR and genotyping

Tissue samples were collected from a total of 410 individuals of *P. homarus* from 16 locations during 2011–2013 throughout the distribution range of the species (Fig. 1, Table 1). The species has previously been reported rarely from the NE Australian coast and New Caledonia, but we were unable to acquire any specimens of this species from those locations over the course of this project. Muscle tissue samples were preserved in 70% ethanol until subsequent DNA extraction. Genomic DNA extraction and mtDNA Control Region (mtCR) and COI amplifications were as described previously [25, 36]. COI fragments were amplified from a representative subset of individuals, with additional sequences obtained from NCBI. Sequences have been submitted to Genbank under accession numbers: CR: KX357386-KX357616, KC625333–KC625469 and KF906454-KF906484; COI: KJ802748–KJ802782.

**Table 1:** Sample locations and sizes for each genetic marker type

Sample Location	Sample Code	Regional Grouping	Sample sizes		
			mtCR	mtCOI	Microsatellites
South Africa	Sa	*P.h.rubellus*	93	5	100
Madagascar	Md		4	3	4
Tanzania	Tn	East Africa	12	5	12
Kenya	K		12	-	12
Oman	Om	NWIO	46	5	44
Iran (Larak)	L		33	11	32
Iran (Chabahar)	Ch		50	10	69
India west (Kannur)	InW	India	9	7^a^	9
India south (Colachel)	InS		34	-	31
Sri Lanka^a^	SrL		-	9^a^	-
India east (Chennai)	InE		7	11^a^	7
Aceh	Ac	Indonesia	14	-	11
Langkawi	My		18	-	18
Lombok	Lo		25	-	26
Vietnam	Vn	East Asia	20	4	20
Taiwan (South)	Tw		3	4	3
Marquesas Islands	M	Marquesas	15	8	12

^a^mtCOI sequences included from Genbank. Accession numbers are given in Additional file 1: Table A1.6

Genetically similar adjacent locations were grouped into Regions that did not cross putative biogeographic barriers

A total of 14 loci were amplified individually or multiplexed in 10 μl PCR reactions. Details of loci, and PCR and sequencing conditions are provided in Additional file 1 in Supporting Information. Each PCR and Genescan run included positive and negative controls to prevent cross contamination and to estimate the genotyping error rates. Null allele frequencies, stuttering, and large allele dropout were checked using Micro-Checker 2.2.3 [37].

### Statistical analyses

Aligned and edited 618 bp sequence fragments of mtCR (using Geneious vR9 [38]), were used for further analysis. A median-joining haplotype network [39] was constructed using mtCR and COI sequences in Network with star contraction [40]. After preliminary analysis, samples from adjacent locations with small sample size that were not significantly different genetically, and did not cross proposed biogeographic barriers, were pooled together for further regional analysis, but also compared with analyses from unpooled samples (Table 1). Gene (haplotype) and nucleotide (л) diversities, Tajima’s D [41] as well as Fu’s F [42] tests and analysis of molecular variance (AMOVA) were calculated using Arlequin version 3.5 [43]. The most likely clusters of genetically similar individuals were estimated from mtCR data using a Bayesian approach in the program Baps v.6 with varying numbers of populations (k) [44].

Microsatellite alleles were sized in Geneious using the microsatellite plugin version 1.4.0 [38]. Of the 14 loci initially genotyped, 9 were retained for further population analysis following quality control analysis, taking regard of reliability, interpretability, and fit of genotype distributions to null expectations. Measures of variability for each locus analysed are reported in Additional file 1: Table A1.2. Analysis of Molecular Variance (AMOVA), and pairwise F_ST_ comparisons between populations and regions were carried out with Arlequin 3.5 [43], Genepop [45] and GenAlEx [46]. Principal coordinate analyses (PCoA) of pairwise genetic distances were undertaken in GenAlEx to display the genetic relationships among sampled populations. Genalex was used to estimate allele frequency, effective number of alleles, observed and expected heterozygosity and fixation index. Allelic richness and gene diversity was analysed in Fstat 2.9.3 [47]. The number of private alleles in each region were adjusted for sample size with a rarefaction approach implemented in Adze [48]. Spatial genetic discontinuities were determined using the Bayesian clustering algorithm Structure [49] either using or excluding a priori locations and groupings (see Additional file 1 for detailed methods). The Benjamini and Hochberg [50] method was used to adjust the family-wide false discovery rate in microsatellite and mtCR sequence data. Additional analysis parameters are provided in Additional file 1.

The ratio of male to female gene flow (m_m_/m_f_) between the two lineages was estimated from the differences between mtDNA Ф_ST_ and nDNA F_ST_, assuming neutral divergence ([51] eq. 7c).

The time to most recent common ancestor (TMRCA), was calculated from the COI data using the Bayesian MCMC approach implemented in Beast 2.4.2 [52]. To enable as direct comparisons as possible with divergence dates calculated in Iacchei et al., [53], the same COI calculation parameters were used, including the 1.39% per lineage divergence rate in a strict clock model. Unfortunately, there are no confirmed divergence times of closely related species on which to base a confident prior. Thus, although there may remain some imprecision in the dates calculated, we can be confident that they are directly comparable with the estimated dates presented in the related studies we discuss. Specific parameters and further details are again provided in Additional file 1.

Gene flow between the *P. h. rubellus* and *P. h. homarus* lineages were estimated in the isolation-with-migration model using IMa2 [54] on the mitochondrial and microsatellite data. Estimates were made independently from the mtDNA and nDNA data sets, as they differed substantially, using search parameters detailed in Additional file 1.

## Results

### Mitochondrial DNA

The mitochondrial control region was highly polymorphic with high haplotype diversity (mean 0.99) (Additional file 1: Table A1.1). Nucleotide diversity was moderate (mean 6.9%), and was significantly lower in the Marquesas (2.1%) (Additional file 1:Table A1.1). Tajima’s D values were negative for most of the locations (Additional file 1: Table A1.1), except for the sample from Marquesas Islands, although not significantly so. The Fu’s F values were also negative for most samples, including the Marquesas.

**Fig. 2:**
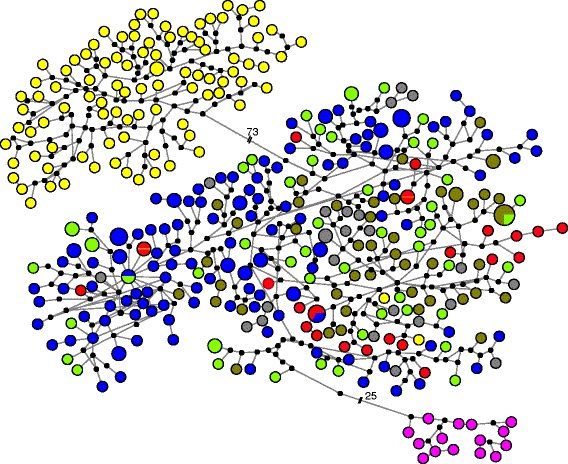
Network of relationships among mtCR haplotypes. Size of coloured pies represent relative frequency. *Black dots* represent un-sampled intermediate haplotypes, one base change distant. Numbers with hashes indicate the number of base changes along that lineage. Colours represent collection regions, and match those of Fig. 1: yellow – South Africa (*P. h. rubellus*), red – East Africa, blue – North-West Indian Ocean (NWIO), light *green* – India, olive *green* – Indonesia, *grey* – East Asia, *pink *– Marquesas

The haplotype network (Fig. 2) showed high haplotypic diversity, with very few haplotypes being shared among locations, but some shared within sampling locations. The network revealed little phylogeographic structure, except for the distinct divergent clades of *P*. *h. rubellus* (South Africa and Madagascar) and samples from the Marquesas Islands. (For a detailed phylogenetic tree of all haplotypes, see Additional file 1: Fig. A1.1). Northwest Indian Ocean (NWIO) haplotypes were found only in restricted parts of the network, in comparison to haplotypes found in other Indian Ocean and West Pacific locations.

The mtCOI analysis of a representative subset of individuals enabled direct comparisons with other studies of *P. homarus* and other species [27, 53, 55]. The haplotype network (Additional file 1: Fig. A1.2) revealed very similar patterns of genetic variation across the distribution of *P. homarus* as seen in the mtCR, but with lower haplotype diversity. Further tests of population subdivision on the mtCOI data (not shown) revealed very similar patterns to those shown in the mtCR analyses presented below, but with less discriminatory power (overall Ф_ST_ = 0.596, *P <* 0.001).

Overall, the AMOVA test of mtCR sequence variation showed a very high level of differentiation among all samples (Ф_ST_ = 0.71, *P <* 0.00001). Hierarchical analysis of all 17 locations grouped into seven regions (areas over which no significant pairwise site differences occurred, and that did not cross proposed biogeographic barriers, Table 1) revealed high levels of differentiation among regions (Ф_RT_ = 0.59, *P <* 0.001), but little divergence among locations within each region (Ф_SR_ = 0.01, n.s.). Excluding the divergent *P*. *h. rubellus* and Marquesas clades also resulted in a significant AMOVA test among the remaining five “central” regions (Ф_ST_ = 0.09, *P <* 0.001).

Analyses of pairwise differences between the sampled regions revealed where the main mtDNA divergences lie within the species (Table 2). As expected, the S. African (*P. h. rubellus*) and Marquesas regions are highly significantly different from all other regions. In addition, all the remaining five “central” regions are significantly different from each other, apart from the Indonesia-E. Africa and Indonesia-E. Asia pairwise comparisons (Table 2). The NWIO region was the most divergent of the “central” regions. Within each of the sampled regions, there were no location pairs that were significantly different from each other (Additional file 1: Table A1.5). Notably, there was no significant difference between *P. h. rubellus* samples from S. Africa and samples from Madagascar (Ф_ST_ = 0.01, *P* = 0.32).

**Table 2:** Pairwise regional differentiation. Above diagonal, mtCR differentiation (Ф_ST_). Below diagonal, microsatellite differentiation (F_ST_)

	S.Africa	E.Africa	NWIO	India	Indonesia	E.Asia	Marquesas
S.Africa		**0.7544**	**0.7831**	**0.7289**	**0.7587**	**0.7356**	**0.7702**
E.Africa	**0.027**		**0.1473**	**0.0410**	0.0099	**0.0337**	**0.6767**
NWIO	**0.012**	**0.012**		**0.0657**	**0.0657**	**0.1509**	**0.6562**
India	**0.027**	**0.010**	**0.009**		**0.0364**	**0.0379**	**0.5350**
Indonesia	**0.033**	**0.022**	**0.019**	0.000		0.0216	**0.6435**
E.Asia	**0.028**	0.003	**0.009**	0.002	0.012		**0.5969**
Marquesas	**0.105**	**0.096**	**0.082**	**0.083**	**0.089**	**0.092**	

All values significant at *P* < 0.05; values remaining sig. After FDR correction in bold

**Fig. 3:**
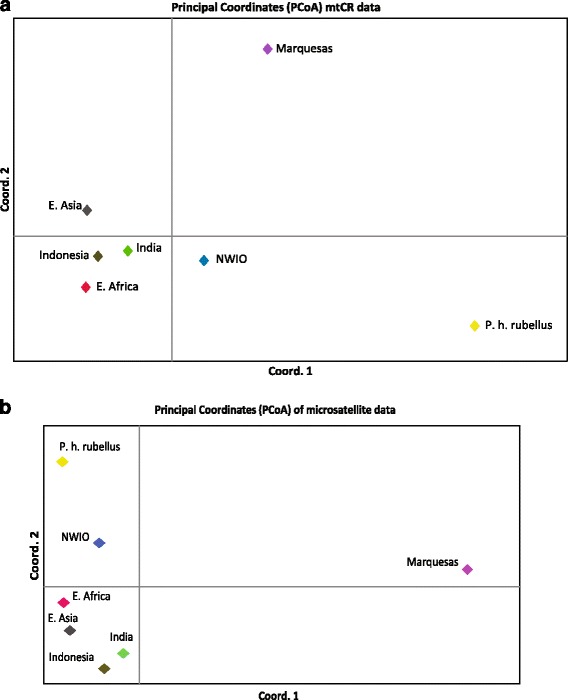
Principal Coordinates Analysis (PCoA) plot of genetic relationships among sampling regions, based on (**a**) pairwise mtCR divergences (Ф_ST_) and (**b**) pairwise microsatellite divergences (F_ST_)

The mtDNA pattern of relationships among regions is best represented in the PCoA of pairwise Ф_ST_ values (Fig. 3a). This shows that *P. h. rubellus* (from S. Africa and Madagascar) is the most divergent region, with the Marquesas also highly divergent. Of the five “central” regions, Indonesia and E. Asia are the most similar. The significantly different mtDNA geographic clusters are also highlighted by the Baps analysis (Additional file 1: Fig. A1.4a). An analysis not using the geographic groupings gave a very similar result. This highlights that, apart from *P. h rubellus* and Marquesas regions, the NWIO region is the most distinct.

A Beast analysis of divergence times between the mtCOI lineages, showed that the *P. h. rubellus* lineage diverged approximately 2.9 million years ago (Ma) (CI: 2.2–3.7), while the Marquesas lineage diverged around 1.25 Ma (CI: 0.9–1.6) (see Additional file 1: Fig. A1.5). In this analysis, the next most closely related species, *P. ornatus*, was estimated to have diverged around 6.8 Ma (CI: 5.4–8.2), which closely matches the previous estimate of approximately 7 Ma [56].

### Microsatellite DNA

Of the nine most reliable microsatellite loci included in the final analyses, all loci exhibited relatively high diversity, with high numbers of alleles per locus (mean *Na* = 15.6) and high heterozygosity (mean *He* = 0.83) (Additional file 1: Table A1.2). Deviation from neutral expectations was observed only in the Marquesas population, where a positive inbreeding coefficient indicated fewer heterozygotes than expected (*F* = 0.115, *P* < 0.05 after correction for false discovery rate).

Overall, the AMOVA test of microsatellite variation showed a significant, but much lower level of differentiation among all samples compared to the mtCR (F_ST_ = 0.035, *P <* 0.001), even after correction for high heterozygosity (F’_ST_ = 0.115, *P <* 0.001) [57]. Hierarchical analysis of all 17 locations grouped into seven regions (see Table 1) revealed significant differentiation among regions (F’_RT_ = 0.189, *P <* 0.001), but also significant differentiation among locations within each region (F’_SR_ = 0.168, *P <* 0.001). Excluding the divergent *P. h. rubellus* and Marquesas clades also resulted in a significant AMOVA test among the remaining five “central” regions (F’_RT_ = 0.097, *P <* 0.01).

Analyses of pairwise differences between collection regions revealed where the main microsatellite divergences lie within the species (Table 2). Again, the Marquesas region is highly significantly different from all other regions, but the S. African (*P. h. rubellus*) region is less significantly diverged. Somewhat surprisingly for a recognised distinct subspecies, *P. h. rubellus* does not exhibit any fixed allelic differences. In contrast to the mtCR results, of the remaining five “central” regions, only the E. Africa and NWIO regions are significantly different from the other regions (Table 2). Within regions, there were several location pairs that were significantly different from each other. In particular, Oman was significantly different from the Iranian samples (F_ST_ = 0.026 *P <* 0.001) (Additional file 1: Table A1.5). Other significant fine-scale differences among locations were not consistent among loci, and these are likely to be artefacts due to small sample size. Again, there was no significant difference between *P*. *h. rubellus* samples from S. Africa and Madagascar (F_ST_ = 0.004, *P* = 0.33).

The microsatellite pattern of relationships among regions is again well represented in the PCoA of pairwise F_ST_ values (Fig. 3b). Unlike the mtDNA analysis, this shows that the Marquesas is by far the most divergent region, with *P. h. rubellus* (from S. Africa and Madagascar) much less so in comparison. Of the five “central” regions, Indonesia and India are the most similar, with the NWIO the most divergent of these. The significantly different microsatellite geographic clusters are also highlighted by the Structure analysis, which shows the *P. h. rubellus* and Marquesas samples to be distinct (Additional file 1: Fig. A1.4b, with optimal k of 5). An analysis excluding the use of a priori locations and regions gave a very similar result.

**Fig. 4:**
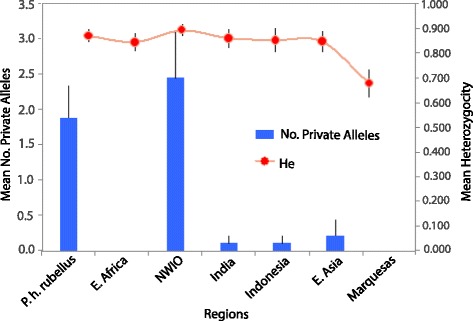
Microsatellite allelic patterns across regions for Mean He – expected heterozygosity, and Mean No. private alleles (found only in that region)

A comparison of the various measures of nuclear microsatellite diversity within each region shows that both the *P. h. rubellus* and NWIO populations contain significant numbers of private alleles (Fig. 4), indicating that they may be more genetically divergent than indicated by the F_ST_ analyses. Heterozygosity values highlight the fact that the Marquesas contain a significantly lowered diversity (Fig. 4). However, tests for the impact of a bottleneck (undertaken in the software Bottleneck, [58]) were not significant.

A Mantel test on the microsatellite data showed an isolation by distance pattern (R^2^ = 0.826, *P <* 0.01), but this pattern was not significant for mtCR data (R^2^ = 0.24, *P* = 0.21), or if the samples from the Marquesas and *P. h. rubellus* populations were excluded (mtCR: R^2^ = 0.0002, *P* = 0.46; microsatellites: R^2^ = 0.0362, *P* = 0.31).

The ratio of male to female gene flow (m_m_/m_f_) between the lineages was estimated from the differences between mtDNA Ф_ST_ and nDNA F_ST_ [51]. Using F_ST_ gave a ratio of 54.2, while using the adjusted F’_ST_ gave a ratio of 3.6. The same ratio calculated between the Marquesas and adjacent populations was 1.1 (0.4 using F’_ST_). The considerable difference in mtDNA and nDNA gene flow between the lineages *P. h. rubellus* and *P. h. homarus* was highlighted by Bayesian estimates from the isolation-with-migration model in IMa2. Maximum likelihood estimates of gene flow from mtDNA peak at zero (m_o>1_ (migration from *P. h. homarus* to *P. h. rubellus*) = 0.0003, 95%HPD 0.0–0.53; m_1>0_ (migration from *P. h. rubellus* to *P. h. homarus*) = 0.0003, 95%HPD 0.0–0.47), while estimates from nDNA were significantly greater than zero (m_o>1_ = 0.51, 0.11–0.59; m_1>0_ = 0.26, 0.16–0.58). (Additional file 1: Table A1.6).

## Discussion

A more extensive sampling of individuals, locations and genetic loci confirms our previous findings of high levels of diversity within and among regions of *P. homarus* [25, 36]. The analyses of mtCR and microsatellite variation provide further evidence that *P. h. rubellus* and the Marquesas population are strongly diverged from the remainder of the species, but low differentiation among centrally-located samples. The differences between the mtDNA and nDNA patterns of divergence of these peripheral populations enable a closer examination of the evolutionary processes driving and maintaining their divergence.

### *P. h. rubellus* (south African) lineage

There is still strong evidence to support a distinct sub-species status for *P. h. rubellus* [25]. This sub-species has a distinct morphological form, is substantially geographically isolated on the extreme of the *P. homarus* distribution, is characterized by belonging to a unique mtDNA lineage that is considerably diverged from the remainder of the species, and is distinct at the ITS-1 nuclear locus [25]. However, the microsatellite results indicate that, surprisingly, there is considerably less nuclear than mtDNA divergence between these sub-species than under neutral expectations [59]. They have very divergent mtDNA lineages (16% K2P for mtCR, compared with 4% divergence of the Marquesas lineage), and a substantial number of private microsatellite alleles, yet only small mean microsatellite allele frequency differences, resulting in significantly larger than expected differences between mtDNA Ф_ST_ and nDNA F_ST_. The *P. h. rubellus* lineage is less divergent than the Marquesas population in its microsatellite DNA, even though it is far more divergent in its mtDNA.

It is known that there is currently some overlap in the distribution of the distinct *P. h. rubellus* and *P. h. homarus* forms along the north-eastern coast of South Africa and Mozambique, with the *homarus* form decreasing in frequency southward [60]. It is also known from previous work that some degree of hybridization occurs between the two forms, as a very small number of individuals with the *rubellus* morphological form have been found to carry the *homarus* mtDNA [25, 61]. Given the geographically highly demarcated boundary to the divergent mtDNA lineages, and the very limited sharing of mtDNA among morphological forms, it is clear that there are very low levels of mtDNA gene flow between lineages. In contrast, the microsatellite results suggest relatively high nDNA gene sharing between these lineages. There are several potential explanations for this phenomenon, and all of them may be relevant to the mechanisms that initiate and maintain marine species divergence and biogeographic boundaries in the Indo-Pacific.

Potentially, the discrepancy in divergence of mtDNA and nuclear microsatellite alleles could be due simply to stochastic lineage sorting with random fixation of mtDNA alleles and/or insufficient time for nuclear alleles to diverge in frequency [62–64]. However, this is unlikely here, given the ratio of mtDNA/nDNA divergence and estimated gene flow, and the extreme contrast in the divergence ratio between *P. h. “brown”* (in the Marquesas) and *P. h. rubellus*. This pattern is close to expectations for neutral divergence in *P. h. “brown”,* which has a mtDNA lineage 4% divergent, no private microsatellite alleles, a mtDNA Ф_ST_/nDNA F_ST_ ratio of 0.59/0.09 and an estimated ratio of male to female gene flow (m_m_/m_f_) [51] of 1.1. In contrast, this pattern is significantly divergent from neutral expectations in *P. h. rubellus,* which has a mtDNA lineage 16% divergent, and a high proportion of private microsatellite alleles, but the mtDNA Ф_ST_/nDNA F_ST_ ratio is 0.75/0.02 and the estimated ratio of male to female gene flow (m_m_/m_f_) is 54.2. This departure from neutral expectations is highlighted by the difference between mtDNA and nDNA estimates of gene flow from the isolation-with-migration model. It is unlikely that either neutral divergence with no gene flow, or high levels of current gene flow in both males and females would leave behind the pattern seen in *P. h. rubellus*.

Given the deep mtDNA divergence, it is more likely that the *P. h. rubellus* lineage diverged allopatrically (or parapatrically) at around 2.9 Ma, and it is only recently that the distributions of the two lineages have begun to overlap, leading to secondary contact and introgression [65, 66]. On initial allopatric divergence, it is not uncommon to see rapid divergence of a new mtDNA lineage, and slower divergence of nDNA alleles, depending on effective population size [59, 62]. Upon secondary contact, there are several possible factors that may have eroded the nDNA divergence more rapidly than the mtDNA divergence, but still maintained lineage divergence through a degree of reproductive isolation [67]. Firstly, marked differences between the sexes in migration behaviour, especially related to breeding, is commonplace among spiny lobsters [68–70], although this seems unlikely to have sufficient impact on a broad scale. Alternatively, asymmetric developmental fitness of hybrids may exist, whereby crosses of one type (i.e., *homarus* males with *rubellus* females) are substantially less fit, resulting in asymmetric male gene flow but restricted female gene flow. The fact that only *rubellus* father/*homarus* mother hybrids have so far been reported may lend some support to this hypothesis [25]. Another possibility is that of a frequency-dependent male preference in both subspecies for conspecific mating. This would result in fewer cross-species matings with the rare female subspecies. Each of these scenarios would result in greater male than female gene flow between lineages. A final possibility (discussed further below) is that of strong environmental selection on differentially adapted phenotypes. The distribution of *P. h. rubellus* extends the furthest south of any *P. homarus* population, across the recognised biogeographic boundary separating warm tropical from cooler subtropical waters along the south-westerly progress of the Agulhas current [71]. If *P. h. rubellus* did diverge allopatrically in the past in this region, there is every likelihood over a substantial period of time that it would adapt to the cooler temperatures of its range. If so, the resulting differential selection along this coast on the two lineages could maintain their largely non-overlapping parapatric distributions, and the degree of reproductive isolation evident today. Further exploration of this potential adaptation is worth pursuing.

*P. h. rubellus* divergence likely originated with the advent of modern glacial cycles at the beginning of the Pleistocene ~2.5 Ma, when a relatively warm, stable interval of global climate transitioned to cooler, high-amplitude cycles, curtailing a warm-water corridor around southern Africa, and intermittently reducing connectivity along this coastline [72]. There is evidence of local adaptation to cooler waters in some other species in this region (*Nassarius* snails [73], *Hymenosoma* crabs [74, 75]), and extensive evidence of ongoing biogeographic barriers that separate tropical, subtropical, warm-temperate and cool-temperate waters along this coast [75]. The breakdown of physical separation between the two lineages likely arose through population expansion during one or more inter-glacial cycles.

Regardless of the exact mechanisms, the data are clear that *P. h. rubellus* likely diverged about 2.9 Ma (CI: 2.2–3.7), that there is a surprising discrepancy in mtDNA and nDNA divergence between these lineages, and that, despite apparently high recent nDNA gene flow, distinct lineages are able to persist in parapatry. This highlights that marine lineages may still be able to maintain divergence despite substantial gene flow, a matter of considerable interest in marine speciation and biogeography [19, 76].

### Marquesas lineage

Like *P. h. rubellus,* the Marquesas population (*P. h. “brown”*) is also a peripherally isolated unique mtDNA lineage, but the data suggest that very different processes have led to its origin and maintenance. Unlike *P. h. rubellus*, its mtDNA lineage is only slightly divergent, and appears to have diverged only around 1.25 Ma (CI: 0.9–1.6). Its mtDNA diversity represents just a small subset of the total diversity present in the neighbouring *P. h. homarus* populations. The nDNA pattern in this population is one of reduction in allelic diversity, but with no unique alleles (unlike *P. h. rubellus*). As such, it may best be regarded as an isolated and distinct population rather than a distinct subspecies, although it does appear to have a distinct morphology [28].

Since its colonisation around 1.25 Ma (CI: 0.9–1.6), likely through a rare period of favourable current flow from the West Pacific (see below), the Marquesas population appears to have diverged genetically through random drift in allele frequencies, with no unique nDNA genotypes. The population appears to have been completely marooned through much of this time, and is now isolated by a very large distance from the main population range of the species (i.e., >7000 km). (The geographically closest recorded specimen is from New Caledonia, >6000 km distant). The lowered levels of mtDNA and nDNA diversity, and positive value of Tajima’s D for the mtCR, all suggest that this population has a reduced effective size, although not sufficient to leave a significant signal of a bottleneck in its microsatellite allele frequencies. This genetic pattern is as predicted by the core-periphery hypothesis: lowered diversity in the periphery compared to central populations, as measured by mtDNA and nDNA allelic diversity and private alleles, and increased genetic divergence of the peripheral population [31].

It has been recognized previously that the Marquesas Islands display a high level of endemism and genetic distinction of its marine fauna [3, 16, 20, 77]. This has been linked to the formation of these islands and local retentive currents [28] and/or prevailing westward currents that now prevent gene flow toward the East Pacific [78].

Thus very different processes appear to have led to the divergence and maintenance of the western and eastern peripheral lineages of *P. homarus*. Further, the eastern periphery matches the expectations of the core-periphery hypothesis, while the western periphery does not.

### Fine-scale population divergences

Examination of mtDNA in *P. h. rubellus* has previously indicated the Madagascan population of this lineage is genetically divergent from the South African population [61]. However, we found no evidence of this distinct gene lineage or any significant divergence in microsatellite alleles in Madagascar. Our sample size from Madagascar may have been insufficient to detect an allele frequency difference, but it should have been possible to detect a distinct mtDNA lineage. Given the distinct lineage observed by Reddy et al. [61] was a COI-like nuclear copy (numt), unlike our COI sequences, it is possible that the divergence identified by this marker is not widespread throughout the mitochondrial or nDNA genomes.

We found strong evidence for reduced gene flow across the distribution of *P. h. homarus,* despite the long larval dispersal period (around 6 months). The NWIO region appears to be genetically isolated (at both mitochondrial and nuclear loci) from regions to the south (Kenya and Tanzania) and to the east (India and beyond). This unexpected degree of isolation observed in both marker types may be explained by the Arab Sea Gyre and east-west monsoon currents [28, 36] which likely act to limit larval transport and gene flow along the north-east African coast and Arabian Peninsula. Larval connectivity with populations further to the east around the Indian subcontinent may also be limited by the significant outflows of turbid fresh water in the vicinity of Karachi (such as the Indus). Similarly, the huge outflows from the Meghna River delta (Brahmaputra outflow) into the Bay of Bengal may help to explain the limited coastal larval connectivity to the east of the Indian subcontinent. Both these regions to the west and east of India appear to be breaks in the natural distribution of *P. homarus*, and may also reflect breaks in larval connectivity. These genetic patterns do not clearly locate the existence of a single mid-Indian Ocean barrier (MIOB), as has been seen in other species [22]. The genetic divergence to the west of India is greater than that to the east, but there is clearly an isolation-by-distance effect along this coastline.

There appear to be few breaks in connectivity between the Indonesian (Aceh and Lombok samples) and East Asian (Vietnam and Taiwan samples) regions, at either mtDNA or nDNA markers. Although our sampling is less intense across this region, the continuous habitat, strong currents, and many stepping-stone islands suggest that connectivity is likely to be strong in this species across this region. Indeed, more intense sampling of this region in the closely related *P. ornatus* (with similar larval duration) has not detected any population differentiation in mtDNA or nDNA [79, 80], and broad sampling of *P. penicillatus* across this region also failed to find any marked genetic differentiation [53, 81].

### Peripheral divergence and biogeographic implications

This study provides further evidence of the ability of marine Indo-Pacific populations to diverge genetically, despite a capacity for extensive planktonic larval dispersal. It is apparent that the levels of gene flow within the species are sufficient to maintain cohesion of the species, except at the peripheries of the distribution range. In the east, the Marquesas population appears to be an isolated remnant of past dispersal, which is now gradually diverging genetically and morphologically. In the west, the great mtDNA divergence and reduced nDNA divergence in the *P. h. rubellus* lineage strongly suggests past allopatric divergence of this lineage, with more recent secondary contact and introgression. The evidence that nDNA gene flow between the lineages is now much higher than mtDNA gene flow, suggests that a partial reproductive barrier has developed during their allopatric isolation, and that this barrier may act through a combination of male mating preferences, and ecological selection on differentially adapted phenotypes. Such a scenario may also be a common feature behind some of the marine biogeographic boundaries commonly observed across the Indo-Pacific.

A similar situation is seen in the closely related *P. ornatus*, where a very distinct mtDNA lineage has been found in the WIO [80]. However, in that species the mtDNA barrier appears to have started breaking down, and this lineage has now spread much more widely eastwards at low frequency. It is possible that this species experienced a similar allopatric divergence history in the WIO as *P. h. rubellus*, but that a behavioural reproductive barrier did not develop in *P. ornatus* before secondary contact and introgression occurred. *P. ornatus* exhibits its greatest mtDNA lineage boundary further north than *P. homarus* (north of Kenya). Another related species, *P. penicillatus*, has its most divergent Indian Ocean mtDNA lineage in its NWIO population, specifically in the Red Sea [53, 82], which itself has now been recognised as being a common biogeographic province [8]. The one factor in common among all these genetically divergent *Panulirus* populations is that they all lie at the extreme western periphery of their species’ distribution. Thus it seems that peripheral populations in the WIO may have a high probability of forming divergent lineages and perhaps species, even if there is potential for long-distance larval dispersal.

Recent studies in other diverse species have also highlighted the importance of the WIO as a location of unique marine lineages and biodiversity. A number of finfish species have been identified as having divergent populations in the WIO [15, 22, 83], and a similar pattern is now also being described in several invertebrate species in the same region [84–86]. Therefore, there appears to be considerable scope for phylogeographic and biogeographic boundaries to be present in the WIO, which has only recently been recognised [15]. As further studies are undertaken in the WIO, we propose that additional common biogeographic barriers will be found, and it appears highly likely that the WIO in general will exhibit higher levels of population divergence than the Indo-Polynesian province to the east for widespread species. We also suggest that the potential mechanisms of population divergence described for *P. homarus* in this study may also apply more widely in this region.

Analysis of mtDNA divergence times in *P. penicillatus* revealed that the East Pacific lineage of that species diverged approximately 1.5 Ma (0.8–2.31), while the NWIO Red Sea lineage diverged only about 0.7 Ma (0.4–1.1) [53]. Similar calculations on the mtDNA divergences in *P. homarus* show that the *P. h. rubellus* lineage likely diverged around 2.9 Ma (2.2–3.7), while the Marquesas sub-population diverged around 1.25 Ma (0.9–1.6). Identical calculations on the *P. ornatus* mtDNA data estimate that the East African lineage diverged around 0.66 Ma [80], and it does not appear to have a peripherally isolated eastern lineage. It is clear from these estimates that there is some, but not complete, overlap in time and place where genetic lineages diverged in these species with similar biology. Around 0.7 Ma, both *P. ornatus* and *P. penicillatus* had peripheral WIO populations that genetically diverged. Around 1.5 Ma, both *P. penicillatus* and *P. homarus* had peripheral Eastern Pacific populations that genetically diverged. The *P. h. rubellus* lineage is by far the oldest divergence among this group, and appears to pre-date these divergences by several million years, but coincides with the estimated date of most common divergence across the MIOB in a range of fish species [22].

These estimated dates of divergence point toward the possibility of common periods of phylogeographic divergence across the Indo-Pacific, which may help to identify the potential initial drivers of divergence. The dramatic impact of sea-level changes on both sea shelf habitat availability and fluctuations in prevailing currents have previously been highlighted, especially in the Indian Ocean, where coastal shelves are generally narrow [18]. However, the divergences reported here fall well before the most recent sea-level changes (around 0.02 Ma), and thus it is very difficult to accurately align any of these divergences with any specific sea-level events. Intersecting with these long-term temporal changes are the complex seasonal changes in currents and upwelling in the WIO that have been identified [36, 86]. Cowman and Bellwood [87] have alerted us to the changes over time in biogeographic processes that have likely taken place in the Indo-Pacific; “accumulation”, followed by “survival”, “origin”, and then most recently a period of “survival and export” of species. All the *Panulirus* lineage divergences reported here fall into the most recent Pliocene period that they characterise as a period of “survival and export” of species. The present study confirms that, even within the tropical spiny lobsters with enormous larval dispersal potential, this has certainly been a period of “export” of unique genetic lineages at the peripheries of the Indo-Pacific.

## Conclusions

The data presented here show that very different evolutionary processes have led to the initial divergence and long-term maintenance of the western and eastern peripheral lineages of *P. homarus*. The pattern of mitochondrial and nuclear divergence of the western lineage, implicates processes of parapatric isolation, secondary contact and introgression, and suggests possible maintenance through adaptation and behavioural reproductive isolation. In contrast, the eastern lineage appears to have diverged through a rare colonisation event, maintained through long-term isolation, and matches expectations of the core-periphery hypothesis. This may provide an explanation of some of the common evolutionary processes that translate phylogeographic divergence into biogeographic divergence, and continue to generate marine diversity throughout the Indo-Pacific.

## Additional file

Additional file 1: Detailed methodological parameters, additional analyses. (DOCX 11816 kb)
