# Supplementary material for: Mechanisms of peripheral phylogeographic divergence in the indo-Pacific: lessons from the spiny lobster Panulirus homarus
Source: BMC Evol Biol. 2017 Aug 18;17:195. doi: 10.1186/s12862-017-1050-8 (PMC5563042; doi:10.1186/s12862-017-1050-8)
Supplement: Additional file 1: — Detailed methodological parameters, additional analyses. (DOCX 11816 kb) [file 12862_2017_1050_MOESM1_ESM.docx]

**Additional File 1**

Mechanisms of peripheral phylogeographic divergence in the Indo-Pacific: Lessons from the spiny lobster *Panulirus homarus*

Ahmad Farhadi, Andrew G Jeffs, Hamid Farahmand, Thankappan Sarasam Rejiniemon, Greg Smith, Shane D Lavery

**Appendix S1**. Detailed methodological parameters, and additional analyses, including PCR conditions, analysis parameter choices, mtCOI network, mtCR pairwise population divergences, and Beast divergence dating results.

#### **Detailed methodological parameters**

#### Mitochondrial DNA

Measures of mtCR variability for each sampled population are reported in Table A1.1. Previously published COI sequences incorporated into the analyses are listed in Table A1.6

#### Microsatellite genotyping

A total of 14 loci (Table A1.2), originally developed for *P. stimpsoni* [1] and *P. ornatus* [2,3] were amplified individually or multiplexed in 10 µl PCR reactions containing 20-50 ng diluted genomic DNA, 1X multiplex type-it master-mix (Qiagen) and 0.2 µM of each forward primer (fluorescently labelled with FAM, VIC, NED or PET) and reverse primers. PCR was carried out on an ABI Veriti^®^ thermal cycler with thermal conditions as follows: 15 min at 95 °C, 32 cycles of 95 °C for 30 s, 60 °C for 50 s, 72 °C for 30 s, with final extension step of 60 °C for 15 min. Genotyping was carried out with 20 times diluted PCR products in 10 µl HiDi formamide along with 0.4 µl of Liz600 size standard (Applied Biosystems) on an ABI3130XL genetic analyser (Applied Biosystems). Measures of variability for each locus analysed are reported in Table A1.3.

#### Structure analyses

Spatial genetic discontinuities were determined using the Bayesian clustering algorithm in Structure ver. 2.3.4 [4], by inferring the highest probable number of genetic clusters present within the dataset with prior knowledge of the individual’s origin. Individuals were placed in K predetermined sub-groups based on their likelihood of belonging to that sub-group, calculated using allele frequencies of multiple loci. K was chosen in advance and ranged from one to 12 and the populations were assumed to be admixed (an individual could belong to any population) in origin. Burn-in and run length were set to 100 000 MCMC (Markov chain Monte Carlo) repetitions and each run was iterated 10 times. MCMC convergence among runs was confirmed using Tracer. The optimal number of clusters, k, was determined with Clumpak.

Beast analyses

The time to most recent common ancestor (TMRCA), was calculated from the COI data using the Bayesian MCMC approach implemented in Beast 2.4.2 [5]. To enable as direct comparisons as possible with divergence dates calculated in Iacchi et al., [6], the same COI calculation parameters were used, including the 1.39% per lineage divergence rate in a strict clock model. This divergence rate was calculated from the 4.7 Myr divergence estimated by Tourinho et al. between the closely related *Panulirus echinatus* and *P. penicillatus,* separated by the Isthmus of Panama. A coalescent tree prior assuming expansion growth was used. The best substitution model, as determined by jModelTest2 [7] and implemented in Beast 2.4.2 was used (TN93 plus gamma). Simulations were run for 20 million generations with sampling every 1000 generations. To ensure adequate mixing, effective sample sizes (ESS) greater than 200 were maintained for all model parameters. Convergence was ensured by using 10 independent runs. Tree files were combined using LogCombiner 1.8.3. They were resampled every 4000 trees after 10% burn-in, giving a total of 50,000 trees. The resulting ultrametric tree is shown in Fig. A1.5.

**Additional Analyses**

MtDNA haplotype phylogenies

A maximum likelihood (ML) phylogeny of the mtCR haplotypes was constructed in PhyML [8], using the best identified nucleotide substitution model (Tr93+G), (Fig. A1.1). A median-joining haplotype network [9] for the COI sequences was constructed using Network with star contraction [10] (Fig. S1.3). A ML phylogeny of the COI haplotypes was constructed in PhyML, using the best identified nucleotide substitution model (Tr93+G), (Fig. A1.3).

Population pairwise differentiation

Pairwise sample population differentiation values are presented for both mtCR (Table A1.4) and microsatellites (Table S1.5). These were calculated in Arlequin, using 1000 randomisations to calculate P values. FDR corrected p-value is 0.009.

Bayesian clustering

The most likely clusters of genetically similar individuals were estimated from mtCR data using a Bayesian approach in the program Baps (Fig. A1.4a). Spatial genetic discontinuities in the microsatellite data were determined using the Bayesian clustering algorithm in Structure (Fig. A1.4b).

The ratio of male to female gene flow

The ratio of male to female gene flow (m_m_/m_f_) between the two subspecies was estimated from the differences between mtDNA Ф_ST_ and nDNA F_ST_ [11]. This assumes equal sex ratios (supported by ecological data; [12], neutral divergence of mtDNA and nDNA genes following a population split, and the attainment of equilibrium conditions. Non-equilibrium conditions would underestimate the m_m_/m_f_ ration. The pairwise measures of mtDNA Ф_ST_ (0.754) and nDNA F_ST_ (0.027) between the *P. h.  rubellus* and *P. h. homarus* were used in eqn 7c from Hedrick et al., [11]. To account for potential bias in nDNA F_ST_ from the high heterozygosity of the microsatellite loci, the adjusted F’_ST_ (0.25) was also used for comparison [13].

Estimates of gene flow from the isolation-with-migration model

Unidirectional estimates of gene flow between *P. h. rubellus* and *P. h. homarus* subspecies were made using IMa2. As estimates from mtDNA and nDNA data diverged considerably, they were analysed separately for each data type. Optimum parameters were determined after several runs, ensuring that there was sufficient mixing, with effective sample sizes greater than 50, and no significant autocorrelations. The parameters -q250 -m0.6 -t60 -b1000000 -l1000000 -hfg -hn20 -ha0.96 -hb0.9 were used for the mtDNA data, and -q150 -m0.6 -t40 -b1000000 -l1000000 -s123 -hfg -hn20 -ha0.96 -hb0.9 for the nDNA.

Table A1.1. MtCR diversity indices for *P homarus* sample collections. Neighbouring locations were pooled to ensure all samples had N>15 for valid comparison.

| Location | N | π± s.d | H± s.d. | Tajima’s D  (p-value) | Fu’s F  (p-value) |
| --- | --- | --- | --- | --- | --- |
| South Africa | 97 | 0.049± 0.0231 | 0.999 ± 0.004 | -1.469* (0.040) | -24.00* (0.000) |
| Tanzania- Kenya | 24 | 0.034 ± 0.0172 | 0.992± 0.0150 | -1.193 (0.099) | -4.797 (0.039) |
| Oman | 46 | 0.030 ± 0.0142 | 0.996± 0.005 | -1.458* (0.046) | -22.20* (0.000) |
| Larak | 33 | 0.038 ± 0.0189 | 0.994 ± 0.009 | -0.922 (0.169) | -8.773* (0.008) |
| Chabahar | 50 | 0.031 ± 0.0147 | 0.993± 0.006 | -1.487* (0.034) | -17.28* (0.002) |
| India | 50 | 0.052 ± 0.0250 | 0.996 ± 0.005 | -1.281 (0.105) | -14.785* (0.001) |
| Aceh- Langkawi | 31 | 0.038 ± 0190 | 0.989 ± 0.012 | -1.441 (0.053) | -4.363 (0.064) |
| Mattaram | 25 | 0.037 ± 0.0189 | 1.000 ± 0.011 | -1.380 (0.072) | -10.401* (0.000) |
| Vietnam-Taiwan | 23 | 0.050± 0.0257 | 0.996 ± 0.014 | -0.808* (0.02) | -4.700 (0.41) |
| Marquesas | 15 | **0.021*** ± 0.0112 | 1.000 ± 0.024 | 1.140 (0.914) | -5.437* (0.013) |
| Overall/mean | 394 | 0.069±0.0021 | 0.999 ±0.000 | -1.030 (0.17) | -11.685* (0.015) |

N, sample size; π, nucleotide diversity; H, haplotype diversity; s.d., standard deviation; Tajima’s D, values of Tajima’s measurement of diversity (Tajima, 1989) and its probability in parenthesis; Fu’s F, values of measure and its probability in parenthesis.

Table A1.2. Microsatellite loci amplified, with measures of their diversity.

| Locus | Repeat type | Allele size range | PIC | Mean Number of alleles | H_exp_ | F (fixation index) |
| --- | --- | --- | --- | --- | --- | --- |
| Orn5 | Tetranucleotide | 142-202 | 0.792 | 23 | 0.816 | -0.010 |
| Orn11 | Tetranucleotide | 134-220 | 0.918 | 27 | 0.924 | -0.071 |
| Orn12 | Tetranucleotide | 228-348 | 0.949 | 44 | 0.952 | -0.017 |
| Stim6 | Dinucleotide | 100-166 | 0.961 | 56 | 0.964 | -0.056 |
| Stim7 | Dinucleotide | 164-226 | 0.961 | 54 | 0.964 | -0.044 |
| Stim19 | Dinucleotide | 100-164 | 0.961 | 54 | 0.964 | -0.092 |
| Stim18 | Trinucleotide | 136-175 | 0.857 | 19 | 0.872 | 0.078 |
| Por731 | Dinucleotide | 80-150 | 0.961 | 73 | 0.964 | -0.096 |
| Por218 | Dinucleotide | 234-300 | 0.936 | 51 | 0.941 | -0.074 |
| Stim5 | Trinucleotide | 194-232 | 0.737 | 11 | 0.764 | Excluded |
| Orn8 | Trinucleotide | 120-700 | 0.998 | 130 | 0.990 | Excluded |
| Orn16 | Tetranuclotide | 133-188 | 0.684 | 12 | 0.721 | Excluded |
| Orn17 | Tetranucleotide | 264-284 | 0.484 | 6 | 0.533 | Excluded |
| Orn17-2 | Tetranucleotide | 332-348 | 0.490 | 5 | 0.542 | Excluded |

Table A1.3. Microsatellite diversity indices for *P homarus* sample collections. N: number of alleles, Na: no. of private alleles, Ne: effective number of alleles, I: Shannon index, A_R_; allelic richness, H_O_: observed heterozygosity, H_E_: expected heterozygosity, F: fixation index.

| Region |  | N | Na | Ne | I | A_R_ | H_O_ | H_E_ | F |
| --- | --- | --- | --- | --- | --- | --- | --- | --- | --- |
| South Africa | Mean  SE | 101.67  1.190 | 20.89  2.085 | 10.19  1.645 | 2.43  0.17 | 7.90  2.63 | 0.81  0.040 | 0.87  0.025 | 0.074  0.039 |
| E. Africa | Mean  SE | 23.11  0.351 | 12.11  1.486 | 8.42  1.271 | 2.17  0.18 | 7.64  2.54 | 0.81  0.046 | 0.87  0.034 | -0.034  0.020 |
| NWIO | Mean  SE | 140.22  1.656 | 23.11  2.508 | 13.03  2.299 | 2.62  0.19 | 8.58  2.55 | 0.84  0.047 | 0.89  0.025 | 0.057  0.036 |
| India | Mean  SE | 43.11  1.428 | 16.56  2.334 | 10.95  1.921 | 2.39  0.22 | 8.17  2.86 | 0.89  0.035 | 0.86  0.041 | -0.037  0.020 |
| Indonesia | Mean  SE | 50.22  2.737 | 17.67  2.759 | 11.19  2.030 | 2.39  0.27 | 8.09  2.70 | 0.85  0.048 | 0.85  0.049 | 0.002  0.029 |
| E. Asia | Mean  SE | 22.56  0.242 | 13.33  1.965 | 9.55  1.759 | 2.23  0.23 | 7.95  2.65 | 0.86  0.046 | 0.84  0.47 | -0.002  0.045 |
| Marquesas Islands | Mean  SE | 11.33  0.553 | 5.78  1.152 | 4.12  0.781 | 1.39  0.20 | 4.86  1.62 | 0.63  0.106 | 0.68  0.056 | **0.115***  0.135 |
| Overall | Mean  SE | 56.03  5.62 | 15.63  1.02 | 9.63  0.70 | 2.33  0.09 | 8.51  2.83 | 0.82  0.025 | 0.83  0.017 | 0.025  0.022 |

Table A1.4. Pairwise population mtCR differentiation. Phi-st values below diagonal, P values above diagonal. Values significant at P < 0.05 in yellow; values remaining sig. after FDR correction in orange.


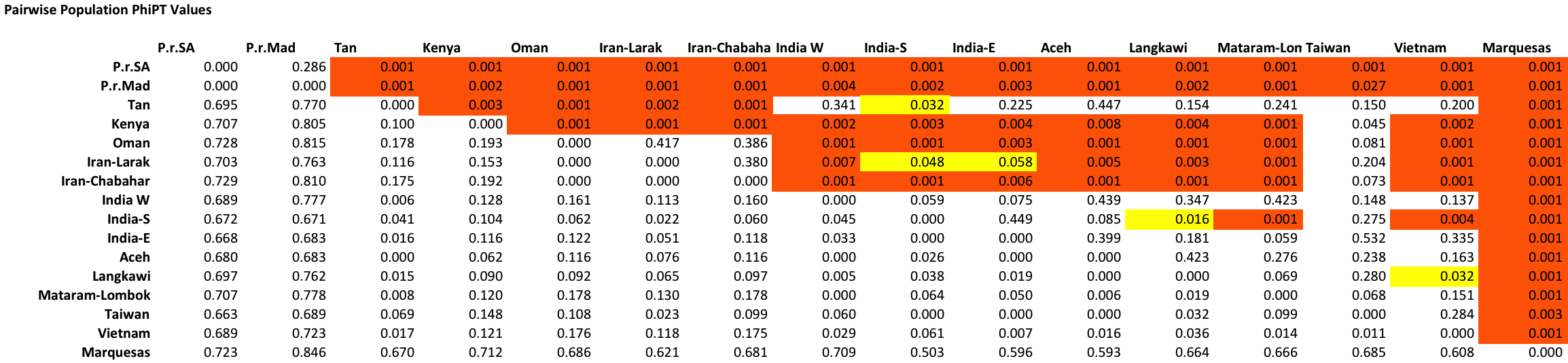


Table A1.5. Pairwise population microsatellite differentiation. F-st values below diagonal, P values above diagonal. Values significant at P < 0.05 in yellow; values remaining sig. after FDR correction in orange.


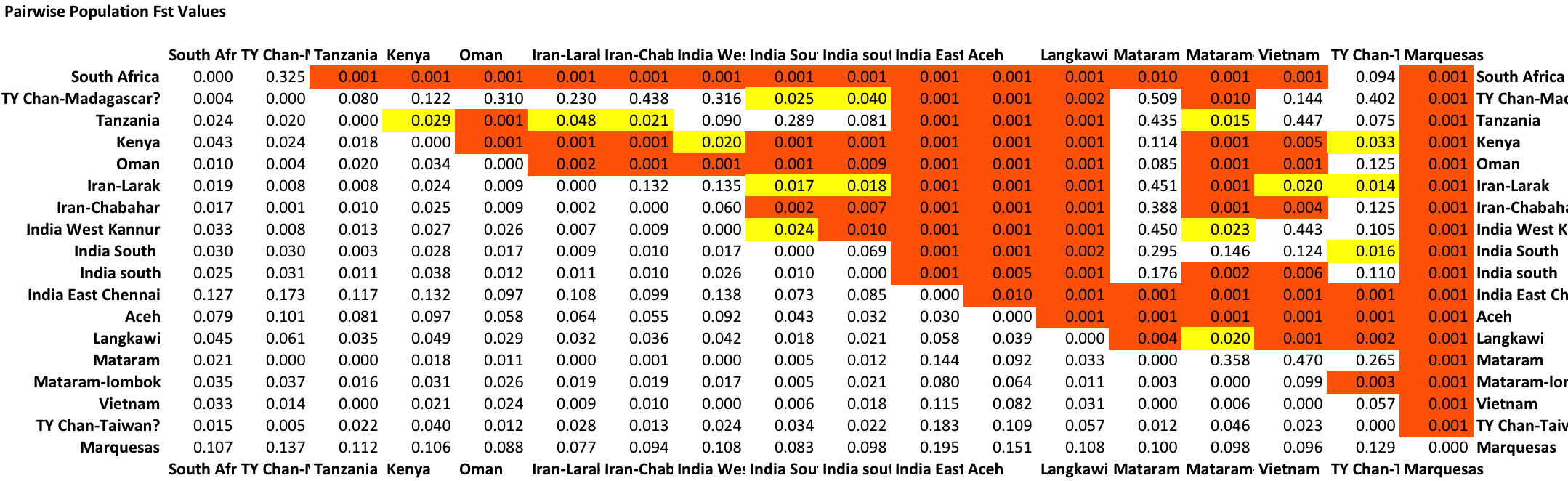


Table A1.6. NCBI Genbank COI sequences used in this study

| No | Accession number | Sample origin |
| --- | --- | --- |
| 1 | JQ229916 | India West |
| 2 | JQ229917 | India West |
| 3 | JQ229918 | India West |
| 4 | JQ229919 | India West |
| 5 | JQ229920 | India West |
| 6 | JQ229921 | India West |
| 7 | JQ229886 | India West |
| 8 | JQ229883 | India East |
| 9 | JQ229885 | India East |
| 10 | JQ229887 | India East |
| 11 | JQ229915 | India East |
| 12 | JQ229910 | India East |
| 13 | JQ229923 | India East |
| 14 | JQ229925 | India East |
| 15 | JQ229884 | India East |
| 16 | JN418937 | India East |
| 17 | JQ229914 | India East |
| 18 | JQ229888 | India East |
| 19 | KC959890 | Sri Lanka |
| 20 | KF548573 | Sri Lanka |
| 21 | KF548570 | Sri Lanka |
| 22 | KF548568 | Sri Lanka |
| 23 | KC959889 | Sri Lanka |
| 24 | KC959891 | Sri Lanka |
| 25 | KF548572 | Sri Lanka |
| 26 | KF548569 | Sri Lanka |
| 27 | KF548576 | Sri Lanka |


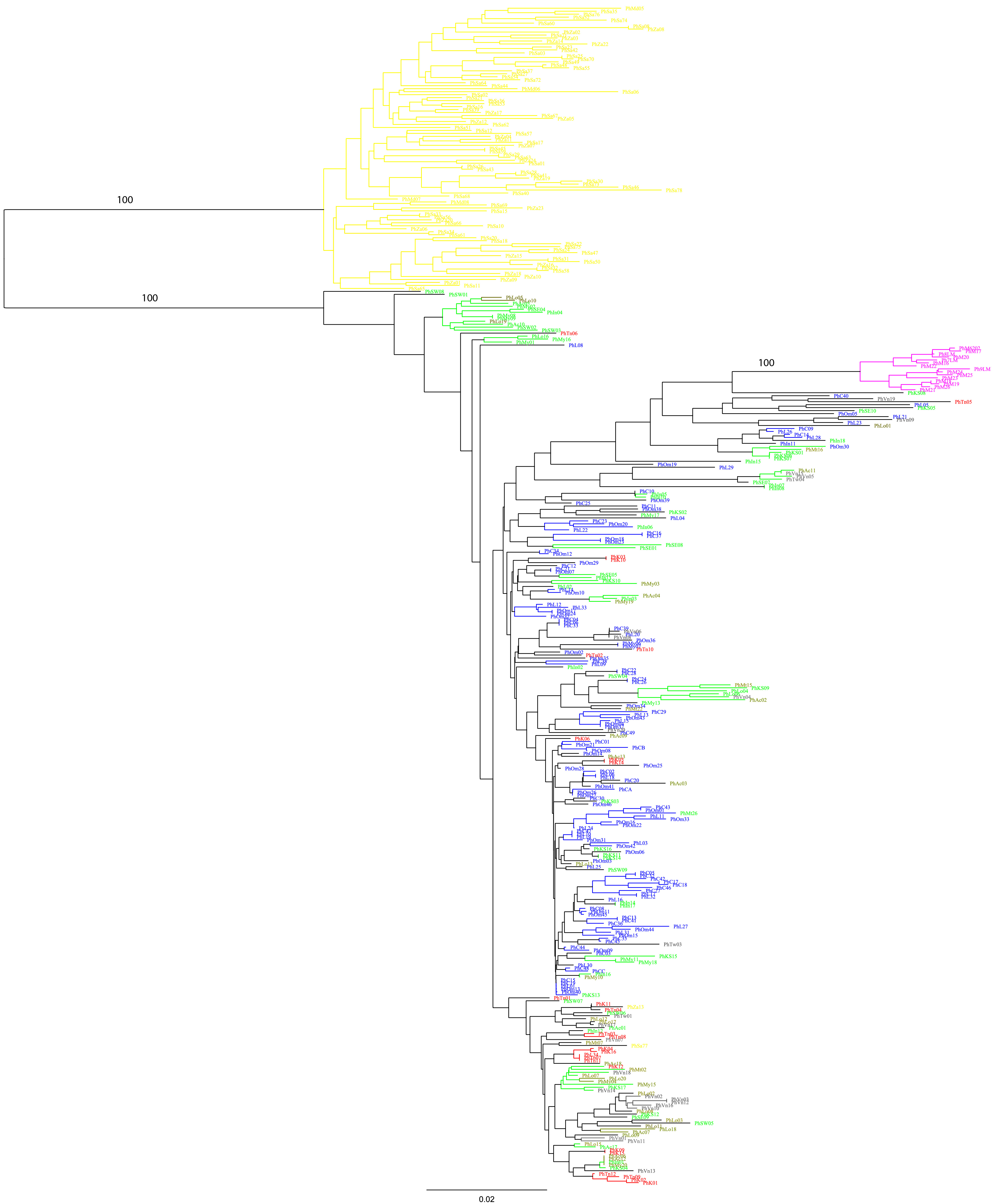


Figure A1.1. ML phylogenetic tree of relationships among mtCR heplotypes. Sequences are coloured according to the same regional colour scheme used throughout: yellow/orange – South Africa (*P*. *h*. *rubellus*), red – East Africa, blue – North-West Indian Ocean (NWIO), light green – India, olive green – Indonesia, grey – East Asia, pink – Marquesas. Bootstrap proportions >50% are shown.


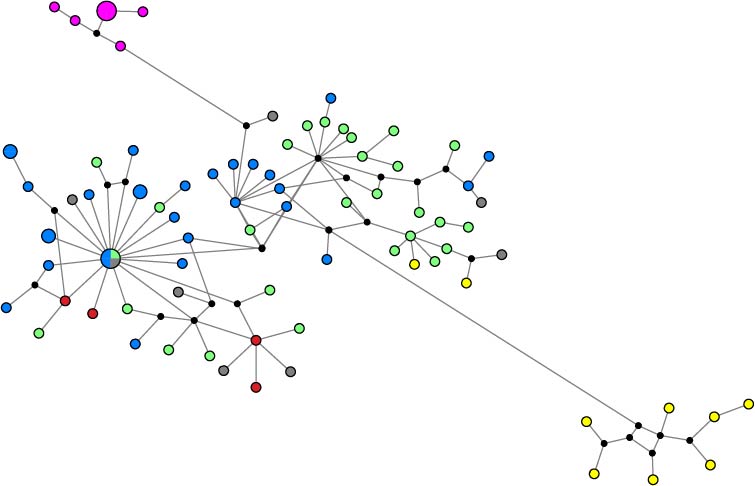


7

30

Figure A1.2. Network of relationships among mtCOI haplotypes. Size of coloured pies represent relative frequency. Haplotypes are coloured according to the same regional colour scheme used throughout.


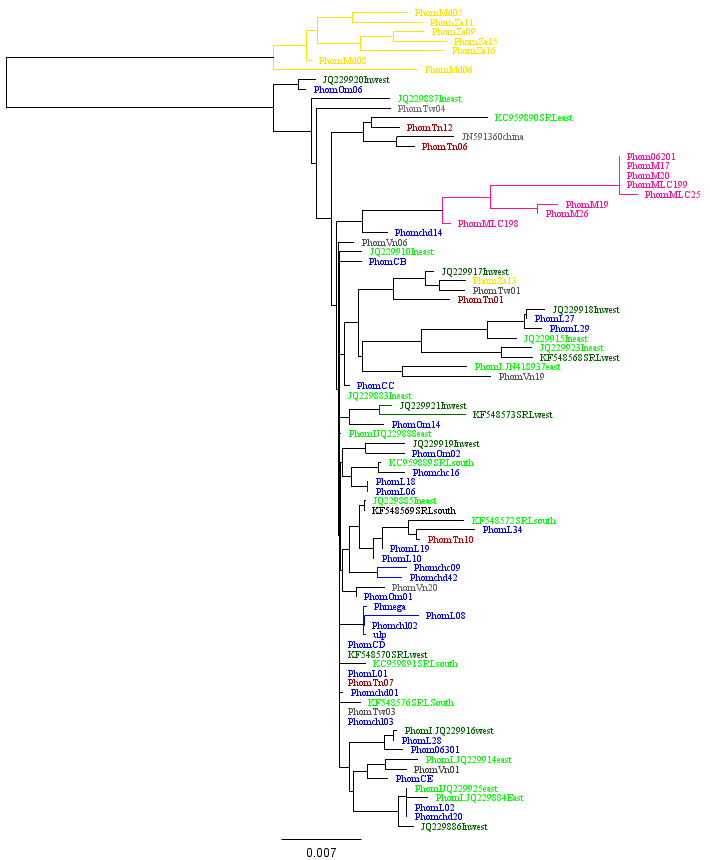


100

100

78

74

84

100

Figure A1.3. ML phylogenetic tree of relationships among mtCR heplotypes. Sequences are coloured according to the same regional colour scheme used throughout. Bootstrap proportions >50% are shown.


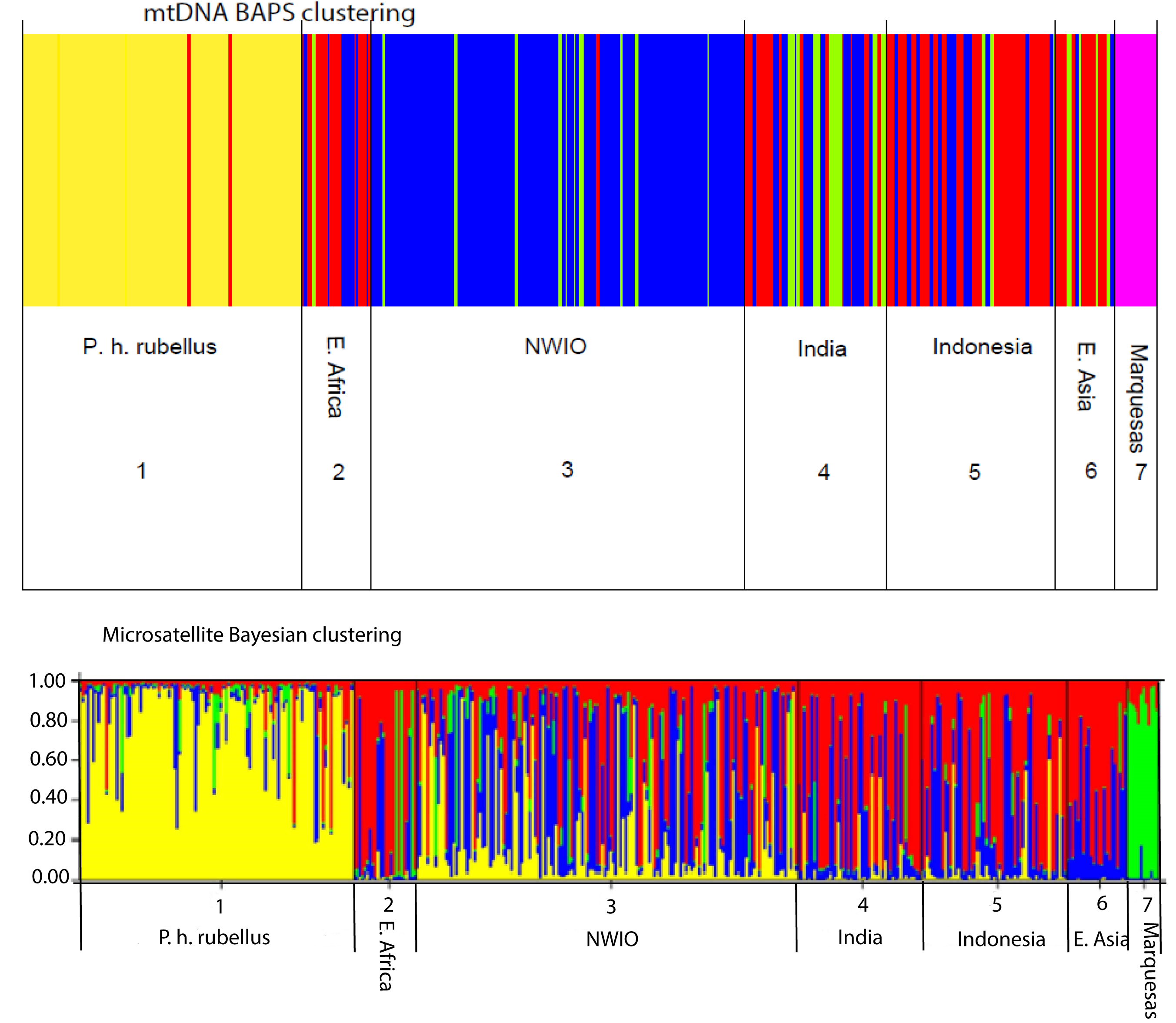


Figure A1.4. Bayesian cluster analysis of individuals into genetically similar groupings, indicated by colour. (a) Baps analysis of mtCR variation, with optimum k=5. (b) Structure analysis of microsatellite variation, with optimum k=5.

Figure A1.5. Maximum clade credibility tree for *P. homarus* mtCOI haplotypes, rooted with *P. ornatus* mtCOI. Compiled from Bayesian MCMC analyses implemented in Beast. Bayesian posterior probabilities are indicated on nodes. Node ages are presented as median node heights with 95% HPD intervals represented by bars.





Figure A1.6. Posterior probability estimates for the extent and direction of migration (as migrations per mutation, *m=M/u*) between *P. h. rubellus* and *P. h. homarus* subspecies from IMa2. mt: mtDNA estimate; n: nDNA estimate; m_o>1_: migration from *P. h. homarus* to *P. h. rubellus*; m_1>0_: migration from *P. h. rubellus* to *P. h. homarus*.

**Additional References**

1. Liu CW, Li JM, Liu L, Guo YS. Screening and genetic diversity analysis of microsatellite markers in Chinese lobster (*Panulirus stimpsoni*). *Yi Chuan* 2010;32:737–43.

2. Liu L, Yang X, Liu CW. Eleven novel polymorphic microsatellite loci in the ornate spiny lobster *Panulirus ornatus* (Decapoda: Palinuridae). *J Genet* 2012;1–3.

3. Dao HT, Todd E V., Jerry DR. Characterization of polymorphic microsatellite loci for the spiny lobster Panulirus spp. and their utility to be applied to other *Panulirus* lobsters. *Conserv Genet Resour* 2013;5:43–6.

4. Pritchard JK, Stephens M, Donnelly P. Inference of population structure using multilocus genotype data. *Genetics* 2000;155:945–59.

5. Bouckaert R, Heled J, Kuhnert D, Vaughan T, Wu CH, Xie D, et al. BEAST 2: A software platform for bayesian evolutionary analysis. PLoS Comput. Biol. 2014;10:e1003537.

6. Iacchei M, Gaither MR, Bowen BW, Toonen RJ. Testing dispersal limits in the sea: range-wide phylogeography of the pronghorn spiny lobster *Panulirus penicillatus*. *J Biogeogr* 2016;43:1032–44.

7. Darriba D, Taboada GL, Doallo R, Posada D. jModelTest 2: more models, new heuristics and parallel computing. *Nat Methods* 2012;9:772–772.

8. Guindon S, Gascuel O. A simple, fast, and accurate algorithm to estimate large phylogenies by maximum likelihood. *Syst Biol* 2003;52:696–704.

9. Bandelt H-J, Forster P, Röhl A. Median-joining networks for inferring intraspecific phylogenies. *Mol Biol Evol* 1999;16:37–48.

10. Forster P, Torroni A, Renfrew C, Röhl A. Phylogenetic star contraction applied to Asian and Papuan mtDNA evolution. *Mol Biol Evol* 2001;18:1864–81.

11. Hedrick PW, Allendorf FW, Baker CS. Estimation of male gene flow from measures of nuclear and female genetic differentiation. *J Hered* 2013;104:713–7.

12. Berry PF. The biology of the spiny lobster *Panulirus homarus* (Linnaeus) off the east coast of southern Africa. Durban: Oceanographic Research Institute; 1971.

13. Hedrick PW. A standardized genetic differentiation measure. *Evolution*. 2005;59:1633–8.
